# A randomised controlled trial of a Mediterranean Dietary Intervention for Adults with Non Alcoholic Fatty Liver Disease (MEDINA): study protocol

**DOI:** 10.1186/s12876-016-0426-3

**Published:** 2016-02-02

**Authors:** Elena S. Papamiltiadous, Stuart K. Roberts, Amanda J. Nicoll, Marno C. Ryan, Catherine Itsiopoulos, Agus Salim, Audrey C. Tierney

**Affiliations:** Department of Rehabilitation, La Trobe University, Nutrition and Sports, Kingsbury Drive, Bundoora, Australia; Department of Gastroenterology, The Alfred Hospital, Commercial Rd, Prahran, Australia; Department of Gastroenterology, 8 Arnold St, Box Hill, Australia; Department of Gastroenterology, St Vincent’s Hospital, Victoria Parade, Fitzroy, Australia; Department of Mathematics and Statistics, La Trobe University, Kingsbury Drive, Bundoora, Australia; Department of Nutrition, The Alfred Hospital, Commercial Rd, Prahran, Australia

**Keywords:** Non-alcoholic fatty liver disease, Mediterranean diet, Fatty liver, Insulin resistance

## Abstract

**Background:**

Non-alcoholic fatty liver disease, the most prevalent liver disease in developed countries, remains difficult to manage with no proven safe and effective pharmacotherapy available. While weight reduction is the most commonly practiced treatment strategy, this is difficult to both achieve and/or maintain in the majority. Furthermore evidence-based dietary recommendations to guide the nutritional management of these patients are lacking. Using a randomised controlled trial design, this study compares the effectiveness of the Mediterranean diet to a standard low fat diet in terms of differences in insulin sensitivity, hepatic steatosis and metabolic outcomes in participants with non-alcoholic fatty liver disease.

**Methods:**

Ninety four eligible patients who have non-alcoholic fatty liver disease and who are insulin resistant, will be randomised into either a Mediterranean or low fat diet group for a 3 month intervention period. Insulin sensitivity will be measured on peripheral blood using Homeostatic Model Assessment and liver fat content quantified using Magnetic Resonance Spectroscopy. Both arms will consist of three face to face and three telephone call follow up consultations delivered by an Accredited Practicing Dietitian. The intervention arm focuses on recommendations from the traditional Mediterranean diet which have been tailored for use in the Australian population The standard arm uses the Australian Guide to Healthy Eating and the Australian National Heart Foundation dietary guidelines. Study recruitment will take place at four major metropolitan hospitals in Melbourne, Australia. Data collection will occur at all face to face reviews including baseline, 6, and 12 weeks. A follow up assessment to measure sustainability will take place at 6 and 12 months. The primary end point is improved insulin sensitivity scores at the 12 week time point.

**Discussion:**

This trial aims to demonstrate in a large cohort of participants with NALFD that a Mediterranean diet independent of weight loss can result in significant benefits in liver fat and insulin sensitivity and that these changes are sustained at 12 months. These metabolic changes would potentially lead to reductions in the risk of chronic liver disease, heart disease, type 2 diabetes and liver cancer.

**Trial registration:**

Australia and New Zealand Clinical Trials Register ACTRN: ACTRN12615001010583.

## Background

Non-alcoholic fatty liver disease (NAFLD) and its progressive form, non-alcoholic steatohepatitis (NASH) are among the most prevalent of liver diseases worldwide. Approximately 20 to 30 % of the adult population has NAFLD, making it the most common liver disease in developed countries [1–3]. The incidence is as high as 50–85 % in people with pre-diabetes and diabetes [4, 5], with diabetic patients having a two to threefold higher risk of dying of chronic liver disease, primarily attributable to NAFLD [6]. In addition, NAFLD has been associated with the occurrence of cardiovascular disease (CVD), independent of the classical risk factors [7]. CVD is the leading cause of death in Australia and globally [8]. An estimated 30 % of patients with NAFLD have NASH, which leads to cirrhosis and its complications including, portal hypertensive bleeding, hepatocellular carcinoma and hepatic decompensation [9]. Insulin resistance is strongly implicated in the development of steatosis (fatty liver) and its progression to NASH. The clinically relevant lesion progresses to cirrhosis in 20–30 % of cases and leads to liver cancer in 2–5 % of cases [10]. Currently no proven, safe, effective therapy exists for NAFLD and NASH [11]. Given the increasing prevalence of these conditions and high incidence of associated metabolic comorbidities, the global burden is expected to steadily increase.

Diet is a modifiable risk factor that can potentially be targeted in both the prevention and treatment of NAFLD. Currently no firm dietary recommendations can be formulated in the management of NAFLD because of the paucity of high quality evidence-based data associated with metabolic outcomes. However current data suggests that patients should aim to achieve a 5–10 % weight reduction, reduce saturated and trans fat, limit fructose, limit intake of simple carbohydrates and substitute with complex carbohydrates such as whole grains, fibre and fruits [12–14]. However, weight loss is neither easy to achieve or maintain and weight loss achieved in research trials is not easily replicated in the clinic or real world settings. As such, there is an urgent need for therapies independent of weight loss.

The health benefits of the Mediterranean Diet (MedDiet) have gained popularity in the scientific literature and lay world with respect to being one of the healthiest diets in the world. Its effects are well researched in high quality studies such as PREDIMED and benefits are attributed to a lower incidence of chronic disease including CVD, obesity, dementia, some cancers, and overall mortality demonstrating its safety, palatability and sustainability [15].

Pilot data to support the shift from current dietary therapy to a novel MedDiet intervention of patients with NAFLD has been published by Ryan et al. in NAFLD patients [16]. This study demonstrated a significant improvement in insulin sensitivity and hepatic steatosis with the MedDiet compared to no change in the low fat diet group.

This study, MEDINA (Mediterranean Dietary Intervention study in Non-alcoholic fatty liver disease) aims to replicate the findings of Ryan et al’s pilot study ina larger cohort of participants with NAFLD in a more translatable environment and with assessment of sustainability. The study aims to investigate the effects of adhering to the MedDiet as compared to a low fat diet. We hypothesise that the MedDiet will demonstrate a potential to reverse insulin resistance and reduce fatty liver and that the benefits will be sustained 12 months post study initiation date with maintenance of the diet. We also aim to explore molecular mechanisms at play in the disease process and the interplay with diet.

## Methods

### Study design and outcome measures

This is a 12 week, multi-centre, parallel, randomised controlled trial of a Mediterranean diet versus a Low Fat Diet (LFD) in patients with NAFLD on insulin resistance, hepatic steatosis and Metabolic Syndrome (MetS) risk factors. Enrolment of 94 participants will occur at four sites across metropolitan Melbourne, Australia: Alfred Health, St Vincent’s Hospital, The Royal Melbourne Hospital and Eastern Health. Both the recruitment and intervention are anticipated to take place over a 24 month period. Participants will undergo stratified randomisation according to gender and diabetes status, into either the intervention; MedDiet or the LFD arm. The primary endpoint is the reduction of one unit of homeostasis model assessment of insulin resistance (HOMA-IR) over the 12 week intervention period. Secondary outcome variables include a reduction in intrahepatic lipid accumulation measured by Magnetic Resonance –Spectroscopy (MR-S), normalisation or improvement of alanine aminotransferase (ALT), lipid profile, liver stiffness as measured by transient elastography (Fibroscan®), inflammatory cytokine markers, quality of life measures, anthropometry and body composition, biomarkers of dietary intake and blood pressure.

### Participant recruitment and eligibility

Participants will be recruited from the outpatient Liver Clinics of four major metropolitan University hospitals via referral from the managing hepatologist of potentially eligible participants for screening. Screening will be performed by a trained researcher.

### Inclusion criteria

Participant eligibility includes those aged >18 years who have a body mass index (BMI) between 20 and 39.9 kg/m^2^. Participants must have a diagnosis of NAFLD determined by routine ultrasound or biopsy; insulin resistance based on a HOMA IR score of >2; and at least one elevated serum ALT level (>20 U/L female, >30 U/L male) during the past 6 months. Eligible participants must have no evidence of another form of liver disease.

### Exclusion criteria

Participants will be excluded if they:

- Refuse or are unable to give informed consent to participate in the study;
- Are Non-English speaking;
- Consume on average >140 g/week of alcohol (men and women);
- Are taking the following medications: immunosuppressants, amiodarone and/or perhexiline;
- Have had a weight change exceeding 5 kg within 3 months;
- Are currently following or anticipated to commence a specialised commercially available weight loss diet and/or program (eg. Light and Easy, Optifast etc.);
- Have a diagnosis of insulin dependent diabetes mellitus or those taking gliclazides;
- Have a HbA1c score exceeding 8 %;
- Have a current or prior history of cardiovascular, cerebrovascular or peripheral vascular disease;
- Have clinically relevant pulmonary, gastro-intestinal, renal, metabolic, hematological, neurological, psychiatric, systemic or any acute infectious disease or signs of acute illness;
- Are women who are pregnant;
- Have psychosocial or gastrointestinal (e.g. malabsorptive conditions e.g. coeliac)
- Have contraindications included bulimia nervosa, substance abuse, clinically significant depression, or current psychiatric care;
- Have had a recent (within 3 months) of change in dose/regime or introduction of vitamin E, C or high dose vitamin D (>3000 IU), fish oil or probiotics.

### Timeline of assessments

All participants will complete a face-to face dietary and lifestyle assessment and consultation, anthropometry and biochemistry at baseline, mid-intervention (6 weeks) and end-intervention (12 weeks). Participants from both groups will also undergo phone call follow up reviews at weeks 2, 4 and 9. All participants will undergo assessment of hepatic steatosis with MR-S and fibrosis with Transient Elastography; Fibroscan® at baseline and end-intervention. Dual-energy X-ray absorptiometry (DXA) scans are voluntary and will assess body composition. All participants will be followed up face to face at 6 and 12 months after study recruitment to determine maintenance and sustainability of the diet administered and subsequent changes in imaging and biomarkers. Please refer to Table 1 for the schedule of measures.

**Table 1:** Schedule of measures

Variable	Instrument	Time point				
Self-reported		Baseline	Mid intervention	End intervention	6 month	12 month
Diet intake	Food Frequency Questionnaire (FFQ), 3- day food diary	X	X	X	X	X
Diet compliance	PREDIMED checklist (low fat or MD)	X	X	X	X	X
Physical activity	Active Australia Questionnaire	X	X	X	X	X
Quality of life	SF-36	X	X	X	X	X
Medications and supplements	Self-report	X	X	X	X	X
Smoking status	Self-report	X				
Ethnicity	Self-report	X				
Medical history	Self-report and clinical records	X	X	X	X	X
Anthropometric and clinical						
Weight, height, waist and hip circumference, neck girth		X	X	X	X	X
Blood pressure		X	X	X	X	X
Whole body composition	Bioelectrical Impedance Analysis (BIA), DXA (subgroup)	X	X *(NO DXA)*	X	X *(NO DXA)*	X
Intrahepatic lipids	MR-S	X		X		X
Liver fibrosis	Fibroscan	X		X		X
Pathology						
	Venous blood sample (fasting)	X	X	X	X	X
	First morning urine sample	X	X	X	X	X
	Buccal swab	X				

### Study procedure

#### Screening assessment

Prospective participants will undergo a thorough screening questionnaire administered face-to-face by a trained researcher. Once considered eligible, participants will undergo computer generated randomisation stratified to gender and diabetes status completed by the statistician. Participants will be allocated into either the LFD or the MedDiet arm of the study and booked in for their initial appointment.

Randomisation will control for equal distribution of key characteristics that may confound between group comparisons and will be assessed by analysis of baseline data.

### Dietary interventions

#### Mediterranean diet

The intervention is based on a traditional MedDiet as described in Keys et al. in the seven countries study and the macro and micronutrient compositions described in previous MedDiet controlled trials [17–20]. The diet is rich in plant based foods including vegetables, whole grains and fruit with the main added fat being extra virgin olive oil. The diet emphasises increased legumes and raw unsalted nut intake and oily fish. Moderate amounts of fermented dairy and poultry with small amounts of red meat and homemade sweets. The diet comprises of 44 % fat (>50 % monounsaturated), 36 % carbohydrate and 17–20 % protein and up to 5 % alcohol. The diet was designed to be easy to follow and sustainable with an ad libitum approach focusing on what should be consumed rather than what to avoid. All changes to diet and lifestyle will be administered to participants by an Accredited Practising Dietitian (APD) who is able to tailor the diet to cultural and personal preferences through recommendation of nutritionally appropriate alternatives where necessary.

All participants will be provided with written resources specifically designed to explain the MedDiet and how it can be successfully followed and adhered to. These resources include a food pyramid, healthy eating guidelines and tips, meal plans, recipes in the form of; The Mediterranean Diet recipe book by Itsiopoulos (ISBN 9781742610825), a shopping list, ‘no cooking’ meal options and label reading. Participants will also be asked to set individual goals at each session which will be used as a focal point for subsequent phone call follow ups and appointments and will assist with having directed changes and maintaining motivation. Participants will also be supplied with extra virgin olive oil (Cobram Estate©) for the duration of the intervention and some MedDiet specific food items such as nuts (Almond Board Australia), canned legumes and fish (Simplot Australian Pty Ltd and HJ Heinz©) which are aimed at providing practical and convenient examples of what items constitute a MedDiet. Breakfast is also provided on the day of all face to face appointments (Jalna © and Carmen’s ©).

#### Low fat diet

The LFD group will follow the same structure as the MedDiet arm with three face to face consultations at baseline, 6 weeks (mid intervention) and 12 weeks (end of intervention). There will also be the same number of phone call follow ups at weeks 2, 4 and 9. The education and resources provided will be determined by the APD running the consultations and these will involve what would normally be provided in a typical outpatient dietetic consult. This will include based on the Australian Guide to Healthy Eating with an emphasis on portions, low fat options and cooking methods. Participants will also be given a supermarket gift voucher to purchase some of the suggested food items.

Breakfast is also provided on the day of all face to face appointments (Jalna © and Carmen’s ©).

### Outcome measures

The outcome measures collected and their parallel timelines are displayed in Table 1. Patient information including demographic data, smoking status, medication and supplement consumption are taken at baseline and checked at each face-to-face appointment for changes.

### Anthropometry

Weight, height, waist circumference, hip circumference, neck girth and blood pressure will be taken using standard procedures, in duplicates by a trained researcher at all face to face appointments.

Body Composition will be assessed using Seca© bio electrical impedance analysis scales at all face to face appointments. DXA scans using a Hologic© machine will a be used to assess body composition in a consenting volunteer subset of participants.

### Dietary and lifestyle

Three day food diaries including 2 weekdays and 1 weekend, a Food Frequency Questionnaire (FFQ) and the PREDIMED Checklist (a measure of dietary compliance specifically designed to measure key elements of the diet administered for each study arm) will be collected from participants at baseline, mid intervention and post intervention to assess dietary compliance relative to the recommendations provided. The Active Australia questionnaire will monitor changes in physical activity. The Short Form Health Survey (SF- 36) will be used to collect quality of life data. All questionnaires will also be administered at 6 and 12 months to observe any sustained behaviours and thus the ability to maintain the recommended changes and any metabolic benefits.

### Imaging

MR-S images will be taken by a qualified radiographer using a Simmens© MRI machine and will be used as the gold standard to monitor changes in the percentage of intrahepatic lipids in the liver. Following a minimum 2 h fast, Transient Elastography (FibroScan®) will be taken in patients to monitor changes to liver fibrosis throughout the study duration.

### Laboratory

Pathology blood samples will be prepared by the nominated laboratories or researchers according to standard protocol. Once prepared samples will be processed and plasma will be stored in −80 °C freezers at the Department of Gastroenterology, The Alfred Hospital Prahran and the Department of Dietetics and Human Nutrition, La Trobe University Bundoora campus until batch analysis of assays are performed.

Plasma fatty acids and urinary metabolites both represent key markers of dietary compliance and will be processed and analysed at La Trobe University. Peripheral blood mononuclear cells (PBMCs) will be utilised to assess metabolomics investigating the genetic variations in genotype/phenotype and will be processed and analysed using polymerase chain reaction (PCR) at La Trobe University. A buccal swab will also be taken at baseline for all participants to assess the DNA profile of the study population.

### Data management

The key researcher at each site will be responsible for storage of hard copies of coded case report forms and questionnaires which will be in kept in secure filing cabinets. All electronic data will be saved in a password protected database. Participants who are interested in the study results will be flagged and individual and overall data will be disseminated at the conclusion of the study.

### Study integrity

Approval to carry out the study was obtained from La Trobe University, the Alfred, Eastern Health, Royal Melbourne Hospital’s research and ethics committee and ethics review is currently underway at St Vincent’s. Written informed consent will be obtained from all participants. This randomised control trial protocol has been designed with close consideration of the CONSORT guidelines [21]. In addition this study protocol has been reviewed by the La Trobe University Research Focus Area board who provided seed funding for the commencement of this trial.

### Sample size

We aim to recruit 47 participants per arm (94 in total) allowing for a 20 % drop out rate over 12 months. This sample size has been calculated with 80 % power to detect a between-group difference in HOMA-IR of 1.0 unit or more at the end of the 12 week duration, with the assumed standard deviations for control and intervention groups of 0.118 and 0.141, respectively. The assumed mean difference and group-specific standard deviations were calculated based on the following past studies: Ryan [16], Esposito [22], Esposito [23] and Elhayany [24].

### Statistical analysis

Intention-to-treat (ITT) principle will be used to analyse and compare the intervention and control groups. As per ITT principle, for the purpose of statistical analyses, all participants will retain their randomised (allocated) group, regardless of the actual treatment received. T-test will be used to examine the effectiveness of randomisation by comparing baseline characteristics across the two groups. If data are not normally-distributed, nonparametric Kruskal-Wallis test will be used instead.

For quantitative endpoints (e.g., HOMA-IR), linear mixed-effect models will be used to compare the intervention and control group. If normality assumption is not fulfilled, suitable transformation (e.g., log) will be performed on the response variable to ensure the assumption is met. For binary endpoints (e.g., improvement in lipid profile >20 %), mixed-effect logistic regression for longitudinal data will be used. In both cases, data from all participants, including those who withdraw after baseline, will be retained for analyses. When required, potential confounders (e.g., age, BMI) will be adjusted for by including them as additional covariates in the models.

When multiple hypothesis testings are performed (e.g. with SNP data), Holm’s procedure (Holm, 1979) will be used to control the familywise error rate. All statistical analyses will be performed using 5 % significance levels. R version 3.1.1 (www.r-project.org) and STATA version 12 will be used to conduct the statistical analyses.

## Discussion

Currently, there are no safe or effective proven therapies for the treatment of patients with NAFLD or NASH [25]. While diet and lifestyle changes focussing on achieving weight loss are the accepted recommendations for this patient group, weight loss is both difficult to achieve and maintain [26–28]. Despite this there have been a limited number of randomised controlled trials in this patient group and no specific diet has been identified as superior to another [29, 30]. In a cross-over study [16] which forms the pilot data for this trial, patients in the MedDiet arm had significant improvements in insulin sensitivity as determined by a 3-hour hyperinsulinaemic-euglycaemic clamp and a significant reduction (39 %) in hepatic steatosis. In contrast there was no significant improvement in insulin sensitivity and only a 7 % reduction of hepatic steatosis with the low fat, high carbohydrate diet. The changes observed in the intervention group were without a significant reduction in weight [16]. The MedDiet has positive effects on insulin sensitivity, which has been attributed to the high content of bio-active phytochemicals with a range of antioxidant and anti-inflammatory activity [31]. The MedDiet is characterised by a specific fatty acid profile: low in saturated fat (7–8 % of total energy) and high in monounsaturated fat (20 % of total energy). A Spanish study showed that treatment with a balanced diet rich in olive oil contributes to the recovery of the liver from hepatic steatosis [32]. This was achieved by decreasing activation of hepatic stellate cell (decrease hepatic collagen) by monounsaturated fatty acids, which is less susceptible to lipid peroxidation as compared to polyunsaturated fatty acids.

This study aims to assess the MedDiet in NAFLD patients to determine whether this dietary pattern is superior compared to the current recommendations for patients, thus developing a more evidence based therapy that can be translated into the clinical setting. In addition by addressing diet and lifestyle factors there is likely to be secondary prevention of associated chronic diseases such as type 2 diabetes and CVD which are shown to share some pathophysiological similarities with NAFLD.

This study is also one of the first randomised controlled trials assessing the MedDiet in a non-Mediterranean country. Thus, will provide insight into implementation of the dietary principals across various cultures and the feasibility and sustainability of these changes in specific ethnic groups. The novel markers of compliance including food diaries and food frequency questionnaires will also be supported by robust markers such as plasma fatty acids and urinary metabolites. Other lifestyle changes including physical activity will be measured using the Active Australia Questionnaire and will be considered in the assessment of final results.

Although environmental influences such as diet have a well-established association with disease it is important to acknowledge that the interplay of genetics and environment are a widely accepted influence in disease outcomes. The relationship between the genome and nutrition (nutrigenomics) is a growing area of research. Studying nutrition alongside genetics is vital as it is now well established that even small differences in gene sequences can alter the metabolic pathways which make individuals more or less responsive to certain diets [33]. Investigating the genotype in terms of response and non-response to the MedDiet in an exploratory manner enhances the understanding at the genomic and molecular level. By examining the associated phenotype with anthropometric and biochemical indices in a NAFLD population and correlating these to inflammation and markers of insulin resistance may help to identify individuals at greater cardio metabolic risk.

This intervention study has the capacity to provide a cost effective and sustainable therapy for large and increasing patient group which we feel would be highly acceptable and widely applicable at a community level, with the potential to reduce cost burden on society and improve health outcomes for individuals.

## Abbreviations

ALT: alanine aminotransferase
CVD: cardiovascular disease
FFQ: food frequency questionnaire
HOMA IR: homeostasis model of assessment insulin resistance
LFD: low fat diet
MedDiet: mediterranean diet
NAFLD: non alcoholic fatty liver disease
NASH: non alcoholic steatohepatitis
SF-36: short form health survey

## References

[1] Wah-Kheong C, Khean-Lee G (2013). Epidemiology of a fast emerging disease in the Asia-Pacific region: non-alcoholic fatty liver disease. Hepatol Int.

[2] Bayard M, Holt J, Boroughs E (2006). Nonalcoholic fatty liver disease. Am Fam Physician.

[3] Paschos P, Paletas K (2009). Non alcoholic fatty liver disease and metabolic syndrome. Hippokratia.

[4] Ortiz-Lopez C, Lomonaco R, Orsak B, Finch J, Chang Z, Kochunov VG (2012). Prevalence of prediabetes and diabetes and metabolic profile of patients with Nonalcoholic Fatty Liver Disease (NAFLD). Diabetes Care.

[5] Chan WK, Tan AT, Vethakkan SR, Tah PC, Vijayananthan A, Goh KL (2013). Non-alcoholic fatty liver disease in diabetics--prevalence and predictive factors in a multiracial hospital clinic population in Malaysia. J Gastroenterol Hepatol.

[6] Zoppini G, Fedeli U, Gennaro N, Saugo M, Targher G, Bonora E (2014). Mortality from chronic liver diseases in diabetes. Am J Gastroenterol.

[7] Targher G, Bertolini L, Padovani R, Rodella S, Tessari R, Zenari L (2007). Prevalence of nonalcoholic fatty liver disease and its association with cardiovascular disease among type 2 diabetic patients. Diabetes Care.

[8] WHO. Cardiovascular diseases (CVDs). Fact sheet N°317. 2015 [cited 2015 16 Feb].

[9] McCarthy EM, Rinella ME (2012). The role of diet and nutrient composition in nonalcoholic Fatty liver disease. J Acad Nutr Diet.

[10] Abenavoli L, Milic N, Peta V, Alfieri F, De Lorenzo A, Bellentani S (2014). Alimentary regimen in non-alcoholic fatty liver disease: Mediterranean diet. World J Gastroenterol.

[11] Williams CD, Stengel J, Asike MI, Torres DM, Shaw J, Contreras M (2011). Prevalence of nonalcoholic fatty liver disease and nonalcoholic steatohepatitis among a largely middle-aged population utilizing ultrasound and liver biopsy: a prospective study. Gastroenterology.

[12] Elias MC, Parise ER, de Carvalho L, Szejnfeld D, Netto JP (2010). Effect of 6-month nutritional intervention on non-alcoholic fatty liver disease. Nutrition.

[13] Abenavoli L, Milic N (2012). Dietary Intervention in Non-Alcoholic Fatty Liver Disease… McCarthy EM, Rinella ME. The role of diet and nutrient composition in nonalcoholic fatty liver disease. J Acad Nutr Diet.

[14] Sofi F, Casini A (2014). Mediterranean diet and non-alcoholic fatty liver disease: new therapeutic option around the corner?. World J Gastroenterol.

[15] Sofi F, Abbate R, Gensini GF, Casini A (2010). Accruing evidence on benefits of adherence to the Mediterranean diet on health: an updated systematic review and meta-analysis. Am J Clin Nutr.

[16] Ryan MC, Itsiopoulos C, Thodis T, Ward G, Trost N, Hofferberth S (2013). The Mediterranean diet improves hepatic steatosis and insulin sensitivity in individuals with non-alcoholic fatty liver disease. J Hepatol.

[17] Itsiopoulos C, Brazionis L, Stoney R, O’Dea K (2011). Can A Mediterranean dietary pattern ameliorate the pro-oxidant effects of diabetes?. Nutr Diet.

[18] Salas-Salvado J, Bullo M, Babio N (2011). Reduction in the incidence of Type 2 diabetes with the Mediterranean diet: results of the PREDIMED-Reus nutrition intervention randomized trial. Diabetes Management.

[19] Itsiopoulos C, Brazionis L, Kaimakamis M, Cameron M, Best JD, O’Dea K (2011). Can the Mediterranean diet lower HbA1c in type 2 diabetes? Results from a randomized cross-over study. Nutr Metab Cardiovasc Dis.

[20] Keys A, Mienotti A, Karvonen MJ, Aravanis C, Blackburn H, Buzina R (1986). The diet and 15-year death rate in the seven countries study. Am J Epidemiol.

[21] Schulz KF, Altman DG, Moher D (2010). CONSORT 2010 statement: updated guidelines for reporting parallel group randomized trials. Ann Intern Med.

[22] Esposito K, Marfella R, Ciotola M, Di Palo C, Giugliano F, Giugliano G (2004). Effect of a Mediterraneanstyle diet on endothelial dysfunction and markers of vascular inflammation in the metabolic syndrome: a randomized trial. JAMA.

[23] Esposito K, Pontillo A, Di Palo C, Giugliano G, Masella M, Marfella R (2003). Effect of weight loss and lifestyle changes on vascular inflammatory markers in obese women: a randomized trial. JAMA.

[24] Elhayany A, Lustman A, Abel R, Attal-Singer J, Vinker S (2010). A low carbohydrate Mediterranean diet improves cardiovascular risk factors and diabetes control among overweight patients with type 2 diabetes mellitus: a 1-year prospective randomized intervention study. Diabetes Obes Metab.

[25] Kaser S, Ebenbichler CF, Tilg H (2010). Pharmacological and non-pharmacological treatment of non-alcoholic fatty liver disease. Int J Clin Pract.

[26] Promrat K, Kleiner DE, Niemeier HM, Jackvony E, Kearns M, Wands JR (2010). Randomized controlled trial testing the effects of weight loss on nonalcoholic steatohepatitis. Hepatology.

[27] Lonardo A, Caldwell SH, Loria P (2010). Clinical physiology of NAFLD: a critical overview of pathogenesis and treatment. Expert Rev Endocrinol Metab.

[28] Franz MJ, VanWormer JJ, Crain AL, Boucher JL, Histon T, Caplan W (2007). Weight-loss outcomes: a systematic review and meta-analysis of weight-loss clinical trials with a minimum 1-year follow-up. J Am Diet Assoc.

[29] Zivkovic AM, German JB, Sanyal AJ (2007). Comparative review of diets for the metabolic syndrome: implications for nonalcoholic fatty liver disease. Am J Clin Nutr.

[30] Yunianingtias D, Volker D (2006). Nutritional aspects of non-alcoholic steatohepatitis treatment. Nutr Diet.

[31] Schroder H (2007). Protective mechanisms of the Mediterranean diet in obesity and type 2 diabetes. J Nutr Biochem.

[32] Hernandez R, Martinez-Lara E, Canuelo A, del Moral ML, Blanco S, Siles E (2005). Steatosis recovery after treatment with a balanced sunflower or olive oil-based diet: involvement of perisinusoidal stellate cells. World J Gastroenterol.

[33] Lairon D, Defoort C, Martin JC, Amiot-Carlin MJ, Gastaldi M, Planells R (2009). Nutrigenetics: links between genetic background and response to Mediterranean-type diets. Public Health Nutr.

